# Social disparities in exposures to bisphenol A and polyfluoroalkyl chemicals: a cross-sectional study within NHANES 2003-2006

**DOI:** 10.1186/1476-069X-11-10

**Published:** 2012-03-06

**Authors:** Jessica W Nelson, Madeleine Kangsen Scammell, Elizabeth E Hatch, Thomas F Webster

**Affiliations:** 1 Boston University School of Public Health, Department of Environmental Health, 715 Albany Street, T4W, Boston, Massachusetts 02118, USA; 2 Boston University School of Public Health, Department of Epidemiology, 715 Albany Street, T3E, Boston, Massachusetts 02118, USA

**Keywords:** Bisphenol A, Polyfluoroalkyl chemicals, PFOS, PFOA, NHANES, Socioeconomic position, Income, Race/ethnicity

## Abstract

**Background:**

Bisphenol A (BPA) and polyfluoroalkyl chemicals (PFCs) are suspected endocrine disrupting compounds known to be ubiquitous in people's bodies. Population disparities in exposure to these chemicals have not been fully characterized.

**Methods:**

We analyzed data from the 2003-2006 National Health and Nutrition Examination Survey. Using multivariable linear regression we examined the association between urinary concentrations of BPA, serum concentrations of four PFCs, and multiple measures of socioeconomic position (SEP): family income, education, occupation, and food security. We also examined associations with race/ethnicity.

**Results:**

All four PFCs were positively associated with family income, whereas BPA was inversely associated with family income. BPA concentrations were higher in people who reported very low food security and received emergency food assistance than in those who did not. This association was particularly strong in children: 6-11 year-olds whose families received emergency food had BPA levels 54% higher (95% CI, 13 to 112%) than children of families who did not. For BPA and PFCs we saw smaller and less consistent associations with education and occupation. Mexican Americans had the lowest concentrations of any racial/ethnic group of both types of chemicals; for PFCs, Mexican Americans not born in the U.S. had much lower levels than those born in the U.S.

**Conclusions:**

People with lower incomes had higher body burdens of BPA; the reverse was true for PFCs. Family income with adjustment for family size was the strongest predictor of chemical concentrations among the different measures of SEP we studied. Income, education, occupation, and food security appear to capture different aspects of SEP that may be related to exposure to BPA and PFCs and are not necessarily interchangeable as measures of SEP in environmental epidemiology studies. Differences by race/ethnicity were independent of SEP.

## Background

Identifying populations that are highly exposed to environmental chemicals is important for protecting public health and preventing health inequalities. Identifying differential patterns of exposure in populations can also provide useful information for hypotheses about possible sources of exposure that, especially for many emerging chemicals of concern, are poorly understood.

This study investigates differences by measures of socioeconomic position (SEP) and race/ethnicity in body burden of two types of chemicals, bisphenol A (BPA) and polyfluoroalkyl chemicals (PFCs). Both are suspected endocrine disrupting chemicals (EDCs) and may alter the normal functioning of hormones and other signaling molecules in the body [1]. BPA is a high volume chemical used industrially to form polycarbonate plastic (PC) and it is present in epoxy resins, including those used as the lining in canned foods [2]. It is an estrogen-like chemical found in some animal studies to disrupt reproductive development, body weight and metabolic homeostasis, and neurodevelopment, and to cause mammary and prostate cancer. Several comprehensive reviews of health outcomes associated with BPA have been published in the last five years [3-7]. PFCs are a class of chemicals used widely in consumer products to impart stain, oil, and water resistance. In particular they are used in food packaging and carpeting and textile treatments [8]. Laboratory studies have found tumors in certain organs and developmental delays in animals exposed to PFCs [9,10], and recent preliminary research in humans reported associations with birth weight, cholesterol levels, and fertility [11-13].

Though BPA and PFCs are ubiquitous in peoples' urine and blood, with U.S. studies detecting them in greater than 90% of people tested [14,15], the specific pathways of human exposure are not well understood. For both chemicals, diet is thought to account for the majority of exposure for most people. In the case of BPA, estimates for adults put the dietary contribution near 100% of total exposure [16,17]; the migration of the chemical from food cans and PC food containers into food may account for most of this, though less-understood exposure routes may also contribute. For PFCs, studies have estimated the dietary contribution as 61% [18], 72% [19], and 91% [20] of total exposure. However, the studies used to develop these estimates are limited in how fully they are able to assess overall human exposure. Recent studies suggest a contribution of indoor air and/or dust to PFC body burdens [21,22].

BPA and PFCs behave very differently once taken into the human body. BPA is rapidly metabolized via glucuronidation, with an estimated urinary elimination half-life in humans of 5.4 hours [23]. A recent study suggests that more accumulation may be occurring than previously assumed, though the half-life is thought to be on the order of days at the most [24]. PFCs, on the other hand, are poorly metabolized, with half-lives of greater than two years in human serum [25,26]. They are thought to bind to proteins in the blood and tissues rather than to lipids, unlike most other persistent organic chemicals [27].

Previous studies have found socioeconomic and racial/ethnic differences in urine and serum levels of BPA and PFCs in a representative sample of the U.S. population. Using data from the 2003-2004 cycle of the National Health and Nutrition Examination Survey (NHANES), Calafat et al. found that urinary BPA concentrations were highest among the lower income group (household incomes less than $20,000), and lowest among Mexican Americans compared to Non-Hispanic Blacks and Non-Hispanic Whites [15]. In contrast, PFC serum concentrations were lower in people with less education (did not graduate from high school), while Mexican Americans had lower levels than other racial/ethnic groups [14]. Differences by SEP and race/ethnicity were not the focus of these studies, and neither included detailed consideration of factors that may explain the disparities.

**Figure 1:**
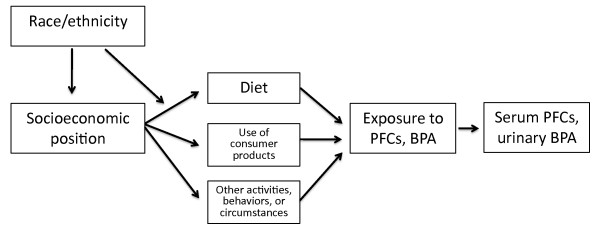
**Conceptual model of the relationship between SEP and race/ethnicity and body burdens of BPA and PFCs**.

SEP and race/ethnicity, in and of themselves, are not likely to explain the differential body burdens of these chemicals; rather, they serve as surrogates for activities, behaviors, or circumstances that may actually contribute to differences. SEP has been defined as "structural locations within society that are powerful determinants of the likelihood of health-damaging exposures and the possession of health-enhancing resources" [28]. Figure 1 presents a framework for conceptualizing these relationships: through several pathways, SEP and race/ethnicity may influence behaviors such as diet and use of consumer products which are sources of exposure to BPA and PFCs. Race/ethnicity is often associated with SEP, and may also be an independent determinant of dietary and other consumer behaviors.

There are numerous ways to characterize SEP; the most commonly used measures are income, education, and occupation. While correlated with one another, each "emphasizes a particular aspect of social stratification" that, in this case, may be more or less relevant to the pathways by which people are exposed to BPA and PFCs [29].

Our study builds on the previous work by Calafat et al. that found opposite associations between measures of SEP and body burdens of BPA and PFCs; one study reported differences by income and the other by education [14,15]. We further investigate these apparent opposite trends by examining relationships between both chemicals and a common set of SEP measures: family income (categorized in four ways), education, occupation, and food security (measured in two ways). Occupation and food security have not been studied before in the general population in relation to both BPA and PFC levels. We also consider the complex relationship between SEP and race/ethnicity, and expand the investigation to an additional NHANES cycle, 2005-2006. This study provides insights into social disparities in exposure to these two types of chemicals, and sheds light on hypothesized sources of exposure.

## Methods

### Study population

We used data from NHANES, an ongoing survey of the civilian non-institutionalized U.S. population conducted by the U.S. Centers for Disease Control and Prevention (CDC). NHANES uses a complex multistage probability sampling design to select participants, and certain racial/ethnic, income, and age groups are oversampled to ensure representativeness [30]. Approximately 5000 participants per year are enrolled, and data are released in two-year cycles. Our study used data from two cycles, 2003-2004 and 2005-2006.

Participants came to a mobile examination center for a physical examination and to provide blood and urine samples, and numerous questionnaires were administered by trained interviewers [30]. Random one-third subsamples of participants had their urine and serum analyzed for environmental chemicals by the National Center for Environmental Health. BPA was measured in urine of participants aged six and older, and PFCs in serum of participants aged 12 and older. The subsamples of participants did not overlap for the chemical analyses. NHANES obtained informed consent from all participants.

### Biomonitoring measurements

Total BPA concentration was measured in urine, and includes BPA parent compound and conjugated metabolites [15]. Measurements were made using solid phase extraction coupled online to high performance liquid chromatography and tandem mass spectroscopy [15]. PFCs were measured in serum using solid phase extraction coupled to high performance liquid chromatography-turbo ion spray ionization and tandem mass spectrometry [14]. The same laboratory techniques were used in both cycles, though limits of detection (LODs) for certain chemicals varied slightly between years. Twelve PFCs were measured in serum. We examined the four PFCs detected in greater than 98% of participants: perfluorooctane sulfonic acid (PFOS), perfluorooctanoic acid (PFOA), perfluorononanoic acid (PFNA), and perfluorohexane sulfonic acid (PFHxS). Values below the LOD were reported by NHANES as the LOD divided by the square root of two.

### Measures of SEP and race/ethnicity

Numerous measures of self-reported SEP were available for participants, assessed through interviews conducted in-person by trained interviewers [31]. We used responses from the following questionnaires: demographics, food security, and occupation (2003-2004 only) [32-36].

Participants reported their annual family income in $5000 increments, with a top category of greater than $75,000. If they refused to answer at this level of detail, they were asked whether their income was less or greater than $20,000. We categorized annual family income in two ways: 1) in four groups, $0-19,999, $20-44,999, $45-74,999, and $75,000 and greater, and 2) in two groups, with a $20,000 cut point, a measure often used in NHANES studies because it maximizes sample size. We also considered the poverty-income ratio (PIR), a ratio of the midpoint of the family income category to the official U.S. poverty threshold as determined by the U.S. Census Bureau, adjusted for family size [35]. A PIR of 1 means that family income is equal to the poverty threshold [37]. We used the following categories: less than 1 (i.e. below the poverty threshold), 1-3, and greater than 3. Finally, we looked at family income adjusted by the square root [38] of family size (available only in 2005-2006 data) or household size (for 2003-2004 data), categorized into quartiles.

Educational attainment was assessed for those aged 20 and older. We used the following categories: less than high school, high school graduate, some college/associate's degree, and college graduate or above.

Data on occupation were available for 2003-2004 only, and for those over age 16. Participants were asked to choose from a list of 41 possible occupational groups for both their current and longest-held job; examples included "teacher," "waiter and waitress," "executive, administrator, manager," and "construction trades" [33]. To categorize occupation, we used an approach that is a hybrid of the U.S. model, which groups jobs by skill, industry, or type (i.e. white collar, service workers, farm workers, blue collar), and the U.K. "work relations" model, which uses 5 categories based on "aspects of work and market situations and of the labor contract" (ranging from managerial/professional to semiroutine/routine) [29]. This hybrid classification system has been employed in previous studies using NHANES data [39]; detail on categories is available in Additional file 1. In our analysis we used information on longest-held occupation.

Food security was measured by NHANES using the U.S. Food Security Survey Module that assesses whether participants and others in their family had adequate food over the last 12 months [32]. Questions included, "were you ever hungry but didn't eat because you couldn't afford enough food?" and "did your child ever skip meals because there wasn't enough money for food?" In 2005-2006, all households were asked the food security questions regardless of income; in 2003-2004, households with incomes over 4 times the poverty threshold were screened out [32,34]. Responses to the individual food security questions were summed by NHANES into an overall food security status variable, and reported as full, marginal, low, and very low. In addition, we looked at whether the participant or a member of their household received emergency food (from a church, food pantry, food bank, or soup kitchen) in the last 12 months.

NHANES assessed race/ethnicity through a series of questions [35]. The participant was first asked whether they consider themselves Hispanic/Latino. They were then asked, "What race do you consider yourself to be?" and could select one or more from a list of fifteen options, including "White," "Black/African American," and "Some other race." Finally, they were asked to choose the one group that best represents their race, with the possible option, "I cannot choose one race." The variable released by NHANES combines these questions and groups people into one of five categories: Mexican American, Other Hispanic, Non-Hispanic Black, Non-Hispanic White, and Other including Multiracial. We also examined whether there were differences among Mexican Americans according to country of birth, since a previous study of polybrominated diphenyl ethers (PBDEs) found important differences in serum concentration by country of origin [40].

### Covariates

We included a small group of covariates in our analyses a priori based on known associations with urine/blood concentrations of BPA and PFCs: age (in categories: 6-11, 12-19, 20-59, older than 60), gender, and in the case of BPA, urinary creatinine, a measure of urinary dilution (continuous variable, mg/dL of urine). We included creatinine as a term in the model rather than using creatinine-adjusted BPA concentrations; creatinine is known to vary by age, gender, and race/ethnicity [41]. As previous studies have reported changes in BPA and PFC body burdens over time, we also controlled for NHANES cycle [14].

We tested to see whether additional variables were acting as confounders; these included time of exam session and total cholesterol (TC, in PFC models only). Participants over age 12 were randomly assigned to either the morning or afternoon/evening exam sessions; those attending the morning session were asked to fast for 9.5 hours, and the latter two for 6 hours [31]. An examination of urinary BPA and reported fasting time in 2003-2004 NHANES data found a decline in BPA concentrations with reported fasting time [24]. Although participants were randomly assigned to exam session time, it is possible that there could be differences in attendance or fasting adherence. TC has been shown to be associated with PFCs in this data set and is likely associated with SEP as well [13].

### Statistical analysis

We compared the different measures of SEP by examining frequency tables of education, occupation, and food security by quartiles of adjusted family income. We analyzed associations between chemical concentrations and SEP and race/ethnicity using multivariable linear regression. Both BPA and PFC concentrations were approximately log-normally distributed; while most individuals had detectable concentrations, the great majority of values were on the low end of the distribution. We thus analyzed both as natural log-transformed continuous variables. We first examined associations with SEP measures separately, controlling for race/ethnicity and the previously-mentioned covariates. Because we wanted to compare different SEP measures, the final study population in the income and food security models consisted of participants who had complete data on all income and food security variables. The sample sizes were smaller for the education and occupation analyses due to the more limited population for which these variables were available (Additional file 2). In this subset of participants we also examined associations with income and food security.

To determine if certain SEP variables were more important predictors than others, we next included multiple SEP variables in the same model. We studied the relationship between SEP and race/ethnicity by comparing results of models with race/ethnicity alone to those that included SEP measures to assess whether this changed the race/ethnicity results. We also considered, separately, interaction by age and gender by including age- and gender-by-SEP terms in the models, and by using stratification.

All regression analyses were performed using the SAS 9.1 Proc SURVEYREG procedure, which takes into account possible correlation between the strata and clusters by which NHANES samples the population. As our intent was to investigate these relationships in a defined population, models were adjusted for relevant covariates instead of using NHANES sampling weights. This adjustment is regarded as a good compromise between efficiency and bias [42].

We present effect estimates for levels of SEP variables and racial/ethnic groups, which represent the percent difference in BPA and PFC concentration for each category compared to the reference group, and their corresponding 95% confidence intervals (CIs). Effect estimates were calculated by exponentiating the natural log-transformed regression coefficients. We assessed statistical significant at the alpha = 0.05 level.

## Results

Of the total NHANES 2003-2006 sample, 5062 participants had BPA measurements and 4214 had PFC measurements; the difference in numbers is due to the fact that PFCs were not measured in 6-11 year-olds. For income and food security measures of SEP, which were available for all age groups, our final sample size was 4739 for BPA and 3953 for PFCs, after excluding those with missing data for the variables of interest (see Additional file 2). The different income measures we studied had different numbers of participants with missing data: family income categorized as less or greater than $20,000 had the fewest missing participants (3%), and adjusted family income and PIR had the most (5%). The final sample sizes for the education analyses were restricted to those older than 20, and for occupation to those older than 16 and in the 2003-2004 cycle.

Table 1 displays unadjusted median concentrations of BPA (creatinine-corrected) and PFCs by covariates, the SEP measures studied, and racial/ethnic groups. Median urinary BPA was highest in children, women, participants in the earlier NHANES cycle, and those with lower incomes. Of the PFCs, PFOS had the highest serum concentrations; median levels were an order of magnitude greater than PFOA, PFHxS, and PFNA. PFCs overall were higher in men than women, and PFOS was highest in the oldest age group and in the earlier NHANES cycle. Differences by income and race/ethnicity were most apparent for PFOS and PFOA, with the highest levels seen in higher income groups and non-Hispanic Whites.

**Table 1:** Distribution of variables and BPA and PFC concentrations by population characteristics

	BPA (n = 4739)		PFCs (n = 3953)				
	**n (%)**	**median (μg/ g creatinine)**	**n (%)**	**PFOA median (μg/L)**	**PFOS median (μg/L)**	**PFNA median (μg/L)**	**PFHxS median (μg/L)**

**Age**							

6 to 11	640 (14)	3.3					

12 to 19	1331 (28)	2.0	1196 (30)	3.7	16.0	0.9	2.1

20 to 59	1865 (39)	2.0	1795 (45)	3.7	17.0	1.0	1.6

> = 60	903 (19)	1.9	962 (24)	4.0	23.5	1.0	1.9

**Gender**							

Female	2390 (50)	2.3	1998 (51)	3.2	15.0	0.8	1.5

Male	2349 (50)	1.9	1955 (49)	4.3	21.2	1.1	2.1

**NHANES cycle**							

2003-2004	2314 (49)	2.6	1929 (49)	3.8	19.8	0.9	1.9

2005-2006	2425 (51)	1.7	2024 (51)	3.7	16.0	1.0	1.7

**Family income**							

$0-$19,999	1360 (29)	2.4	1185 (30)	3.4	16.5	0.9	1.7

$20,000-$44,999	1502 (32)	2.1	1326 (34)	3.7	17.9	0.9	1.8

$45,000-$74,999	924 (19)	2.0	735 (19)	4.0	18.5	1.0	1.8

$75,000 and over	953 (20)	1.9	707 (18)	4.2	19.8	1.1	2.0

**Poverty income ratio**							

PIR < 1	1104 (23)	2.4	869 (22)	3.2	14.4	0.8	1.7

PIR 1-3	1983 (42)	2.2	1742 (44)	3.7	17.9	0.9	1.8

PIR > 3	1652 (35)	1.8	1342 (34)	4.2	20.2	1.0	2.0

**Adjusted family income^a^**							

Quartile 1	1077 (23)	2.5	864 (22)	3.3	15.7	0.8	1.7

Quartile 2	1156 (24)	2.2	1040 (26)	3.6	17.0	0.9	1.7

Quartile 3	1142 (24)	2.1	938 (24)	3.8	18.4	0.9	0.2

Quartile 4	1364 (29)	1.8	1111 (28)	4.2	20.6	1.1	2.0

**Food security status**							

Very low	294 (6)	2.6	216 (5)	3.7	17.6	0.9	1.8

Low	564 (12)	2.3	458 (12)	3.3	14.5	0.9	1.6

Marginal	416 (9)	2.3	350 (9)	3.4	16.3	0.9	1.7

Full	3465 73)	2.0	2929 (74)	3.9	18.8	1.0	1.8

**Emergency food**							

Yes	362 (8)	2.4	291 (7)	3.4	15.0	0.8	1.7

No	4377 (92)	2.1	3662 (93)	3.8	18.2	1.0	1.8

**Education^b^**							

Less than high school	753 (27)	1.9	780 (28)	3.4	18.3	0.9	1.7

High school grad/GED	671 (24)	2.0	686 (25)	4.0	19.1	1.0	1.7

Some college/AA degree	775 (28)	2.1	782 (28)	3.9	18.9	0.9	1.7

College grad or above	566 (20)	1.8	508 (18)	4.1	21.0	1.1	1.8

**Longest occupation^b^**							

Never worked	142 (8)	2.4	142 (9)	3.5	17.4	0.7	2.1

Blue collar, semi-routine	656 (39)	2.3	684 (42)	3.9	19.6	0.9	1.9

Blue collar, high skill	226 (13)	2.5	212 (13)	3.6	22.0	0.9	1.7

White collar, semi-routine	324 (19)	2.5	289 (18)	3.8	19.6	0.9	1.7

White collar & professional	347 (20)	2.3	311 (19)	3.9	21.8	1.0	1.8

**Race/Ethnicity**							

Non-Hispanic White	1985 (42)	2.2	1781 (45)	4.2	19.9	1.0	1.9

Non-Hispanic Black	1226 (26)	2.2	1013 (26)	3.7	19.5	1.0	1.9

Mexican American US born	677 (14)	1.9	469 (12)	3.5	15.4	0.7	1.7

Mexican American Foreign born	488 (10)	1.9	431 (11)	2.6	11.8	0.7	1.4

Other Race/ Multiracial	222 (5)	2.1	142 (4)	3.6	18.6	1.0	2.0

Other Hispanic	141 (3)	2.0	117 (3)	3.8	14.8	1.0	1.7

^a ^Quartiles of adjusted family income were calculated in the overall 2003-2006 population, so the numbers within the PFC and BPA subsets are unequal

^b ^Education includes only those over age 20 (BPA n = 2765, PFC n = 2756), and occupation only those over age 16 and in the 2003-2004 NHANES cycle (BPA n = 1695, PFC n = 1638)

The different SEP measures were related to one another in a predictable fashion: of those who graduated from college, 67% were in the top adjusted family income quartile; 43% of participants who never worked were in the bottom quartile; and 53% and 57% of those with very low food security or who received emergency food, respectively, were in the bottom quartile (Additional file 3). However, there was some discordance across SEP variables. For example, almost 30% of participants with less than a high school education were in the top two income quartiles; the distribution of the occupational categories, particularly the "blue collar, high skill" group, was fairly evenly distributed across income quartiles; and close to 40% of those reporting full food security were in the bottom two quartiles.

### Socioeconomic position

In adjusted regression analyses, urinary concentrations of BPA were inversely related to all four measures of income (Table 2). For example, those in the lowest quartile of adjusted family income had BPA concentrations 27% (95% CI, 15 to 40%) higher than those in the highest income quartile. Though the four family income variables revealed similar patterns, the magnitude of the difference was decreased with the two-category variable. We also saw higher concentrations in those with very low food security, and those who received emergency food. Though we did not see an inverse trend with educational attainment, college graduates had the lowest BPA levels. Results for occupation did not reveal a consistent pattern, though the "blue collar, high skill" group (including vehicle mechanics, construction workers, and members of the armed forces) had higher BPA concentrations. In these and all other models, controlling for exam session did not change the observed associations with SEP or race/ethnicity.

**Table 2:** Percent change in BPA and PFC concentrations by different SEP measures^a^

	BPA (n = 4739)	PFOA (n = 3953)	PFOS (n = 3953)	PFNA (n = 3953)	PFHxS (n = 3953)
	**% change (95% CI)**	**% change (95% CI)**	**% change (95% CI)**	**% change (95% CI)**	**% change (95% CI)**

**Family income**					

< $20,000	**14.3 **(6.2, 23.0)	**-10.9 **(-16.4, -5.0)	**-12.8 **(-17.7, -7.7)	**-11.0 **(-17.5, -4.0)	**-9.7 **(-16.8, -1.9)

≥ $20,000	ref	ref	Ref	ref	ref

**Family income**					

$0-$19,999	**22.8 **(10.6, 36.4)	**-15.9 **(-22.5, -8.7)	**-19.3 **(-24.6, -13.8)	**-17.8 **(-26.5, -8.0)	**-16.1 **(-24.1, -7.3)

$20,000-$44,999	**11.79 **(2.1, 22.4)	**-10.1 **(-15.2, -4.6)	**-11.6 **(-17.1, -5.8)	**-11.3 **(-18.4, -3.5)	**-11.0 **(-19.4, -1.7)

$45,000-$74,999	6.9 (-3.0, 17.9)	-1.3 (-6.5, 4.2)	-5.4 (-11.8, 1.4)	-6.6 (-13.5, 0.8)	-5.3 (-15.1, 5.6)

$75,000 and over	ref	ref	Ref	ref	ref

**Poverty income ratio**					

PIR < 1	**27.0 **(15.7, 39.3)	**-21.2 **(-26.2, -15.9)	**-22.1 **(-26.7, -17.2)	**-16.9 **(-24.0, -9.2)	**-19.0 **(-26.4, -11.0)

PIR 1-3	**16.2 **(7.8, 25.2)	**-13.2 **(-17.5, -8.7)	**-11.0 **(-15.4, -6.3)	**-11.4 **(-17.0, -5.5)	**-13.7 **(-22.3, -4.1)

PIR > 3	ref	ref	Ref	ref	ref

**Adjusted family income**					

Quartile 1	**26.6 **(14.9, 39.5)	**-20.5 **(-26.4, -14.0)	**-21.6 **(-26.3, -16.6)	**-18.0 **(-26.1, -8.9)	**-18.4 **(-25.6, -10.5)

Quartile 2	**18.8 **(6.7, 32.4)	**-16.4 **(-21.0, -11.4)	**-16.4 **(-21.5, -11.0)	**-15.4 **(-21.7, -8.6)	**-17.1 **(-26.1, -7.0)

Quartile 3	**15.3 **(6.2, 25.1)	**-10.9 **(-15.9, -5.7)	**-10.4 **(-15.6, -4.9)	**-10.3 **(-15.9, -4.4)	**-12.1 **(-20.2, -3.3)

Quartile 4	ref	ref	Ref	ref	ref

**Food security status**					

Very low	**19.6 **(5.6, 35.5)	-4.1 (-14.0, 7.0)	-4.7 (-13.6, 5.1)	-5.1 (-17.0, 8.6)	-4.1 (-18.0, 12.3)

Low	3.1 (-8.5, 16.1)	**-9.3 **(-15.5, -2.5)	**-11.0 **(-17.4, -4.0)	**-8.7 **(-14.3, -2.7)	-8.3 (-17.4, 1.9)

Marginal	8.2 (-0.9, 18.2)	-4.2 (-9.6, 1.6)	-4.0 (-10.2, 2.6)	-4.5 (-14.0, 6.2)	-0.9 (-13.5, 13.6)

Full	ref	ref	Ref	ref	ref

**Emergency food**					

Yes	**16.0 **(2.4, 31.4)	**-11.6 **(-18.4, -4.2)	**-14.2 **(-21.0, -6.7)	**-16.5 **(-25.0, -7.1)	-6.5 (-17.4, 5.7)

No	ref	ref	Ref	ref	ref

**Education^b ^**					

Less than high school	6.8 (-7.2, 22.9)	-8.0 (-17.2, 2.1)	-6.5 (-15.1, 2.9)	-5.3 (-14.9, 5.4)	-8.1 (-19.4, 4.9)

High school grad/GED	**17.9 **(3.2, 34.8)	-0.1 (-6.1, 6.3)	-6.3 (-13.5, 1.4)	-5.7 (-13.6, 3.0)	**-9.1 **(-16.8, -0.6)

Some college/AA degree	**16.9 **(0.9, 35.4)	-1.1 (-7.9, 6.3)	-2.9 (-10.7, 5.7)	-8.1 (-16.4, 1.0)	-9.7 (-19.7, 1.5)

College grad or above	ref	ref	Ref	ref	ref

**Longest occupation^b ^**					

Never worked	2.5 (-18.7, 29.1)	-8.1 (-22.5, 9.1)	-9.5 (-23.5, 7.2)	**-24.2 **(-36.9, -9.0)	8.6 (-11.7, 33.5)

Blue collar, semi-routine	6.1 (-6.5, 20.4)	0 (-8.2, 8.9)	-4.5 (-15.1, 7.4)	-4.2 (-13.8, 6.4)	-0.7 (-9.3, 8.8)

Blue collar, high skill	**24.4 **(4.7, 47.7)	-11.7 (-24.4, 3.0)	-5.6 (-19.5, 10.6)	-13.2 (-28.0, 4.7)	1.0 (-16.4, 22.2)

White collar, semi-routine	6.3 (-9.2, 24.4)	3.8 (-7.7, 16.7)	3.0 (-8.3, 15.7)	-5.0 (-15.4, 6.8)	7.8 (-8.0, 26.3)

White collar & professional	ref	ref	Ref	ref	ref

^a ^Models adjusted for NHANES cycle, age, gender, race/ethnicity, creatinine (BPA). Statistically significant results in bold

^b ^Education models include those over age 20 (BPA n = 2765, PFC n = 2756), and occupation models those over age 16 and in the 2003-2004 NHANES cycle (BPA n = 1695, PFC n = 1638)

Results for PFCs revealed an opposite relationship than that for BPA; all four PFCs had strong positive associations with income (Table 2). For PFOA, those in the lowest quartile of adjusted family income had PFC serum concentrations 21% (95% CI, -26 to -14%) lower than those in the highest quartile. As in the BPA analysis, using the two-category income variable attenuated the association, and income measures adjusted for household size resulted in stronger associations with PFC levels. Those who received emergency food had lower concentrations of PFOS, PFOA, and PFNA, as did those with low food security (although we did not see the same strong association with very low food security). Associations between education and occupation and PFC level were weaker than for income, though PFNA concentrations were lower in those who had never worked. When restricted to the subset with information on education and occupation, relationships for income, food security, and emergency food assistance were slightly stronger than in the population overall (data not shown). Similar to exam session, controlling for TC in these and all other PFC models did not affect results.

When multiple SEP measures were included in the same model, adjusted family income remained the predictor of the greatest magnitude and strength for both BPA and PFCs (Table 3). Effect estimates for food security status and use of emergency food decreased when income was added, though, for BPA, regression coefficients remained elevated in the same pattern (but without statistical significance).

**Table 3:** Percent change in BPA and PFC concentrations with multiple SEP variables in the same model^a^

	BPA	PFOA	PFOS	PFNA	PFHxS
	**% change (95% CI)**	**% change (95% CI)**	**% change (95% CI)**	**% change (95% CI)**	**% change (95% CI)**

**1. Adjusted family income + education^b^**	(n = 2765)	(n = 2756)	(n = 2756)	(n = 2756)	(n = 2756)

Adjusted family income quartile 1	**23.6 **(8.7, 40.5)	**-23.8 **(-30.5, -16.5)	**-23.9 **(-30.7, -16.4)	**-22.1 **(-30.8, -12.2)	**-22.7 **(-32.1, -11.9)

Quartile 2	**18.4 **(4.7, 33.9)	**-19.0 **(-24.7, -12.8)	**-18.5 **(-24.4, -12.1)	**-19.3 **(-25.9, -12.0)	**-20.5 **(-29.9, -9.8)

Quartile 3	9.5 (-1.6, 21.9)	**-12.2 **(-18.6, -5.4)	**-11.7 **(-18.1, -4.8)	**-10.2 **(-16.1, -3.9)	**-13.4 **(-21.6, -4.5)

Quartile 4	ref	ref	ref	ref	ref

Less than high school	-2.8 (-15.0, 11.0)	3.6 (-7.1, 15.6)	5.2 (-5.2, 16.8)	6.1 (-4.7, 18.2)	3.6 (-9.4, 18.5)

High school grad/GED	10.6 (-3.1, 26.2)	**8.0 **(1.0, 15.4)	1.1 (-6.9, 9.8)	1.5 (-6.9, 10.7)	-1.4 (-9.7, 7.8)

Some college/AA degree	12.0 (-3.0, 29.4)	4.5 (-2.8, 12.3)	2.5 (-5.8, 11.5)	-3.3 (-11.8, 6.0)	-4.4 (-14.7, 7.0)

College grad or above	ref	ref	ref	ref	ref

**2. Adjusted family income + food security status**	(n = 4739)	(n = 3953)	(n = 3953)	(n = 3953)	(n = 3953)

Adjusted family income quartile 1	**25.5 **(14.3, 37.7)	**-20.8 **(-27.8, -13.1)	**-21.7 **(-27.1, -15.9)	**-17.8 **(-26.2, -8.5)	**-19.0 **(-27.5, -9.5)

Quartile 2	**18.3 **(6.7, 31.1)	**-16.5 **(-21.4, -11.3)	**-16.4 **(-21.7, -10.7)	**-15.3 **(-21.9, -8.1)	**-17.5 **(-26.9, -6.7)

Quartile 3	**14.9 **(5.9, 24.7)	**-11.0 **(-16.1, -5.6)	**-10.5 **(-15.6, -5.0)	**-10.3 **(-15.9, -4.3)	**-12.4 **(-20.6, -3.3)

Quartile 4	ref	ref	ref	ref	ref

Food security very low	10.7 (-2.6, 25.9)	4.9 (-7.2, 18.6)	4.8 (-5.3, 15.9)	2.3 (-10.6, 17.0)	3.8 (-13.2, 24.2)

Low	-3.6 (-13.1, 7.0)	-2.3 (-9.9, 6.0)	-3.8 (-11.1, 4.1)	-2.7 (-8.8, 3.7)	-1.8 (-12.4, 10.0)

Marginal	2.3 (-5.8, 11.2)	1.7 (-4.1, 7.9)	2.1 (-4.9, 9.5)	0.6 (-9.1, 11.4)	4.9 (-8.9, 20.8)

Full	ref	ref	ref	ref	ref

**3. Adjusted family income + emergency food**	(n = 4739)	(n = 3953)	(n = 3953)	(n = 3953)	(n = 3953)

Adjusted family income quartile 1	**24.6 **(13.4, 36.9)	**-19.7 **(-26.1, -12.7)	**-20.4 **(-25.2, -15.4)	**-16.0 **(-24.7, -6.2)	**-18.4 **(-26.1, -9.8)

Quartile 2	**17.8 **(6.2, 30.8)	**-15.9 **(-20.7, -10.8)	**-15.8 **(-20.7, -10.5)	**-14.4 **(-21.0, -7.3)	**-17.1 **(-26.2, -6.8)

Quartile 3	**15.0 **(5.9, 24.8)	**-10.8 **(-15.8, -5.5)	**-10.2 **(-15.4, -4.8)	**-10.1 **(-15.7, -4.0)	**-12.1 **(-20.2, -3.3)

Quartile 4	ref	ref	ref	ref	ref

Emergency food yes	8.3 (-3.7, 21.9)	-5.1 (-13.2, 3.7)	-7.5 (-15.1, 0.8)	**-11.8 **(-21.6, -0.9)	-0.4 (-12.8, 13.8)

No	ref	ref	ref	ref	ref

^a ^Models adjusted for NHANES cycle, age, gender, race/ethnicity, creatinine (BPA). Statistically significant results in bold

^b ^Education models include only those over age 20

### Modification by age and gender

We found some evidence for different effects by age in the results for adjusted family income and food security. Overall, the effect estimates for family income were most consistent in 20-59 year-olds, with a clear trend for BPA and all four PFCs (Additional file 4). For BPA, income was only associated with urinary levels in the younger three age groups; there was no association in the oldest age group. The strong association between BPA concentrations and food security (both very low food security and use of emergency food) was markedly stronger in 6-11 year-olds. Children who received emergency food had BPA levels 54% higher (95% CI, 13 to 112%) than children who did not. This relationship was much smaller in 12-19 and 20-59 year-olds and not evident at all in those over 60. Results were similar in the very low food security group, except that participants over 60 had increased concentrations similar to 6-11 year-olds. For PFCs, the inverse associations by income and food security were most apparent in 20-59 year-olds except for PFHxS, where associations were also strong in those over 60.

We observed fewer differences by gender (data not shown). Very low food security and receipt of emergency food were more strongly associated with BPA concentrations in women than in men. For PFCs, emergency food was more strongly associated in men than in women, whereas the magnitude of the association for family income was greater in women than in men.

### Race/ethnicity and SEP

Tables 4, 5 examines the relationship between race/ethnicity and SEP. In models unadjusted for a measure of SEP, BPA concentrations were lower in Mexican Americans compared to Non-Hispanic Whites (Table 4). The relationship was stronger in Mexican Americans born in the U.S. than in those born elsewhere (Table 5). The association became even stronger when controlling for adjusted family income, indicating that this difference was not mediated by income. When stratified by quartile of adjusted family income, the decrease in Mexican Americans relative to other groups was strongest in the lowest two income quartiles and not evident in the top quartile (data not shown). BPA concentrations in Non-Hispanic Blacks and Whites were similar.

**Table 4:** Percent change in BPA and PFC concentrations by race/ethnicity (with Mexican Americans as one group), with and without control for family income^a^

	BPA	PFOA	PFOS	PFNA	PFHxS
	**% change (95% CI)**	**% change (95% CI)**	**% change (95% CI)**	**% change (95% CI)**	**% change (95% CI)**

	**(n = 4739)**	**(n = 3953)**	**(n = 3953)**	**(n = 3953)**	**(n = 3953)**

**Without control for family income**					

Non-Hispanic Black	4.1 (-4.8, 12.3)	**-14.9 **(-22.2, -6.8)	4.4 (-4.3, 13.9)	**16.0 **(2.4, 31.4)	-6.8 (-22.1, 11.6)

Mexican American	**-12.4 **(-20.8, -2.0)	**-28.8 **(-34.5, -23.7)	**-28.8 **(-34.5, -23.5)	**-24.3 **(-32.6, -15.0)	**-25.7 **(-37.0, -12.4)

Other Race, Multiracial	-7.7 (-21.2, 9.7)	**-18.8 **(-30.5, -5.2)	-9.8 (-22.9, 4.8)	1.1 (-12.5, 16.2)	-4.9 (-23.0, 16.0)

Other Hispanic	2.0 (-13.1, 19.7)	-10.6 (-20.3, 0.3)	**-20.7 **(-29.5, -10.9)	12.0 (-8.3, 36.6)	-22.9 (-40.9, 1.0)

Non-Hispanic White	ref	ref	ref	ref	ref

**With control for family income**					

Non-Hispanic Black	-1.0 (-9.5, 7.0)	**-10.4 **(-18.1, -3.4)	**8.9 **(0, 19.4)	**20.3 **(6.0, 36.4)	-4.0 (-19.7, 16.2)

Mexican American	**-17.3 **(-25.5, -9.9)	**-25.2 **(-30.9, -19.1)	**-25.0 **(-30.7, -18.7)	**-20.5 **(-28.8, -10.8)	**-21.3 **(-33.9, -6.8)

Other Race, Multiracial	-10.0 (-23.4, 5.6)	**-16.5 **(-28.2, -2.8)	-7.2 (-20.5, 7.8)	3.8 (-10.1, 19.9)	-2.0 (-20.8, 19.4)

Other Hispanic	-2.8 (-18.4, 15.0)	-5.3 (-15.6, 6.4)	**-15.8 **(-25.0, -5.4)	17.8 (-3.5, 44.8)	-18.2 (-37.9, 7.8)

Non-Hispanic White	ref	ref	ref	ref	ref

^a ^Models adjusted for NHANES cycle, age, gender, creatinine (BPA), adjusted family income (where noted). Statistically significant results in bold

**Table 5:** Percent change in BPA and PFC concentrations by race/ethnicity (with Mexican Americans as foreign- v.U.S.-born), with and without control for family income^a^

	BPA	PFOA	PFOS	PFNA	PFHxS
	**% change (95% CI)**	**% change (95% CI)**	**% change (95% CI)**	**% change (95% CI)**	**% change (95% CI)**

	**(n = 4376)**	**(n = 3694)**	**(n = 3694)**	**(n = 3694)**	**(n = 3694)**

**Without control for family income**					

Non-Hispanic Black	3.7 (-4.5, 12.6)	**-13.9 **(-21.3, -5.8)	5.3 (-3.0, 14.9)	**15.7 **(2.0, 31.1)	-7.1 (-22.1, 11.4)

Mexican American, Foreign born	-8.6 (-20.5, 5.1)	**-40.2 **(-45.0, -35.0)	**-40.2 **(-45.1, -34.3)	**-25.9 **(-33.9, -17.3)	**-37.1 **(-47.3, -24.4)

Mexican American, US born	**-14.4 **(-23.2, -3.9)	**-16.6 **(-24.7, -7.6)	**-15.9 **(-21.3, -10.1)	**-22.8 **(-34.5, -8.6)	-12.5 (-26.8, 5.1)

Non-Hispanic White	ref	ref	ref	ref	ref

**With control for family income**					

Non-Hispanic Black	-1.1 (-9.2, 8.3)	**-11.6 **(-18.8, -3.0)	9.2 (-0.2, 19.5)	**19.7 **(5.6, 35.8)	-4.2 (-19.7, 15.2)

Mexican American, Foreign born	**-16.5 **(-26.7, -3.9)	**-36.1 **(-41.2, -30.5)	**-35.6 **(-41.0, -29.7)	**-20.5 **(-28.9, -12.2)	**-33.1 **(-44.5, -18.9)

Mexican American, US born	**-18.1 **(-26.8, -7.7)	**-14.4 **(-22.8, -5.1)	**-13.5 **(-19.2, -6.8)	**-20.7 **(-32.5, -6.9)	-10.4 (-25.4, 8.3)

Non-Hispanic White	ref	ref	ref	ref	ref

^a ^Models adjusted for NHANES cycle, age, gender, creatinine (BPA), adjusted family income (where noted). Statistically significant results in bold.

Mexican Americans also had the lowest concentrations of all four PFCs (Table 4). When controlled for income, these differences decreased slightly. Foreign-born Mexican Americans had lower levels of PFCs than those born in the U.S. (Table 5) With PFOA, for example, foreign-born Mexican Americans had serum concentrations that were 40% lower (95% CI, -45 to -35%) than non-Hispanic Whites, whereas the difference in those born in the U.S. was 17% (95% CI, -25 to -8%). This difference by country of origin was less apparent for PFNA. Stratification by adjusted family income revealed that Mexican Americans for the most part had the lowest levels of PFCs across all income quartiles, compared with other ethnicities, with some evidence for slightly stronger decreases in the lowest income quartiles (data not shown). Non-Hispanic Blacks had lower PFOA concentrations compared to Non-Hispanic Whites, but higher PFNA and, to a lesser extent, PFOS levels. These positive associations increased with control for income.

## Discussion

Our findings show that people with lower incomes, who may be more likely to suffer from other disparities in health and exposures, have a greater burden of exposure to BPA. The results for children are especially troubling. Children overall had higher urinary BPA concentrations than teenagers or adults, but children whose food security was very low or who received emergency food assistance - in other words, the most vulnerable children - had the highest levels of any demographic group. Their urinary BPA levels were twice as high as adults who did not receive emergency food assistance. Concerns about health effects from BPA exposure are strongest for young children and neonates because they are still undergoing development [3]. Results for BPA by race/ethnicity, adjusting for income, revealed that Non-Hispanic Whites and Blacks had similar urinary levels, and being Mexican American appeared to be highly protective.

Findings for PFCs revealed differences by socioeconomic position in the opposite direction. Participants with the highest incomes had the highest serum concentrations. We did not see the same vulnerability in younger age groups as with BPA; associations with income were strongest in adults. However, NHANES did not measure PFCs in 6-11 year-olds. While there was some variation by race/ethnicity, Non-Hispanic Whites had the highest levels for two of the four PFCs and being Mexican American again appeared to be protective.

The possible pathways by which SEP is associated with differential exposures to BPA and PFCs may be elucidated by comparing results for the individual SEP variables. Family income was by far the most consistent and important predictor of concentrations; it had a clear dose-response pattern for all chemicals, and remained the strongest when included in models with other SEP variables simultaneously. Conceptually, income reflects access to material goods; a family's current household income is the most specific measure of their immediate financial resources [28]. Thus, income may affect exposure to BPA and PFCs via types of foods consumed or via other consumer products used (or not used) in the home. Past studies that have examined the effect of income, education, and occupation on diet quality have consistently found that income is the most important and strongest predictor of diet [43,44]. Given that diet is assumed to be a major pathway of exposure to these chemicals, differences in food purchasing patterns by income seems one likely explanation for the observed differences.

The literature on specific differences in diet by measures of SEP is large; a review offers this summary of socioeconomic status (SES) and dietary intake: "available evidence suggests that consumption of whole grains, lean meats, fish, low-fat dairy products, and fresh vegetables and fruit was consistently associated with higher SES groups, whereas the consumption of fatty meats, refined grains, and added fats was associated with lower SES groups" [45]. Cost of food is a compelling hypothesis for why this differential exists, as foods with higher energy density are cheaper per amount of energy, but also tend to be nutrient-poor [45]. This is thought to be an important reason why consumption of fresh fruits and vegetables in particular is lower in people with lower incomes. Income is also a strong determinant of where a person lives, and there is a growing body of literature on the lack of access to large supermarkets and ample fresh fruits and vegetables in lower income neighborhoods [46].

This is the first study to examine the relationship between body burdens of BPA and PFCs and two measures of food security as possible proxies for SEP. We conceptualized these variables as representing the intersection of income and dietary behavior, and assumed that those with very low food security, or those who received emergency food, were an especially vulnerable population in terms of accessible dietary options. Particularly in regards to BPA exposure, we hypothesized that they would be more likely to eat canned foods. Recent research indicates that eating canned and packaged foods can contribute to BPA body burdens [47]. We did see associations in the hypothesized direction between BPA and the food security measures; there was a particularly strong signal with very low food security compared to low and marginal food security, and an association of slightly lower magnitude in those who received emergency food. These associations were attenuated when controlling for income, though coefficients remained positive. More striking were the associations between BPA and food security in 6-11 year-olds, which were of the greatest magnitude of any age group. This could be due to greater consumption of foods containing BPA, or the fact that children consume more food per body weight than adults. Though use of emergency food was also associated with PFCs, food security status was not as important a predictor for these compounds.

We saw fewer and less consistent associations between education and concentrations of BPA and PFCs, particularly PFCs. Education is a long-term indicator of SEP, and embodies the transition in SEP from childhood to adulthood [28,44]. In terms of the specific ways in which education may impact exposure to chemicals, it is thought to represent the ability to access and interpret health-related information [28]. With exposure to BPA and PFCs, however, this type of knowledge may not be useful in reducing exposures, as consumers most often do not know that they are being exposed to these chemicals, nor how exposure is occurring. The recent flurry of media and political action around BPA in baby bottles and PC water bottles may be changing this dynamic for BPA, and may explain the slightly stronger associations we observed with education, but increased attention began only in the last few years and is not relevant for the bulk of the study period [48]. Similar to previous studies, we found some discordance between education and current income [44,49]. For BPA, which has a very short half-life, we would be more interested in current and not long-term income, since the foods and products a person purchased in the very recent past directly contribute to urinary levels.

There appeared to be little association between BPA and PFCs and occupation; however, our ability to draw conclusions about these relationships is limited by the fact that we had smaller numbers as data were only available for adults from 2003-2004. We did not see many associations between the compounds and occupation classified into five skill- and work relations-based categories. It seems unlikely that work-related psychosocial stress would affect exposure, though the physical conditions of a workplace could contribute to exposures. Examples include working in an office with new carpeting or furniture that contains PFC precursors [21] or a job in retail that involves handling credit card receipts that contain BPA [50].

The weaker associations observed for education and occupation may also be partly related to the fact that both are measured on the level of the individual, whereas family income and food security are family-level measures [44]. The latter two variables may be more accurate measures of SEP insofar as family purchasing patterns are concerned. This distinction could be important for food purchasing behavior, as it is not clear who in the family (i.e. the participant or some other family member) makes the food shopping decisions.

Our results clearly show differences in BPA and PFC body burdens by measures of SEP that were not explained by race/ethnicity, and vice versa. It is likely that cultural behaviors and patterns are associated with race/ethnicity independent of SEP. The strikingly lower concentrations of both chemicals in Mexican Americans, even after controlling for income, was the most notable result regarding race/ethnicity. This is particularly unexpected for BPA, where Mexican Americans do not fit with the observed pattern of lower income groups having higher urinary concentrations. Mexican Americans and Hispanics have been shown to have higher intake of fruits and vegetables compared to Non-Hispanic Blacks and Whites in different population-based surveys, including NHANES 2003-2004 [51], the 2005 California Health Interview Survey [51], and the 2000 National Health Interview Survey [52]. Eating more fresh fruits and vegetables is likely to be associated with eating less canned foods, which may explain the lower urinary BPA levels seen in Mexican Americans compared to other groups.

In addition, we observed that foreign-born Mexican Americans had markedly lower serum concentrations of PFCs than U.S.-born Mexican Americans, except for PFNA. This is consistent with the fact that PFCs have long half-lives, and exposure from many years past (i.e. when living in Mexico, where exposures may be lower) could impact current serum levels. Similar patterns have been seen for some other persistent lipophilic chemicals [40,53]. For BPA, foreign- and U.S.-born Mexican Americans had similar levels, which makes sense given that BPA has a short half-life, and lower exposures from years past would not matter.

A final aspect of SEP and its relationship with race/ethnicity that must be mentioned is wealth. Wealth can be thought of as the "accumulated assets" of an individual or family, usually in the form of savings, real estate, and inherited items, and represents economic security [54]. While there are no direct measures of wealth in NHANES, differences in wealth by race/ethnicity are reported to be much larger than differences by income; for the same income, the amount of wealth for African Americans and Hispanics has been shown to be much lower than for Whites [28]. Thus, adjusting for income alone may underestimate the real effect of SEP [28], and differences by race/ethnicity may suffer from residual confounding due to inability to adjust for wealth.

Our findings have various practical implications for environmental epidemiology. It is standard in environmental epidemiology studies to include some measure of SEP as a covariate in models. But, it is rare to see a discussion of the rationale for the choice of SEP variable. In many instances, there seems to be an assumption that different measures, particularly income and education, serve as surrogates for the same underlying phenomenon, and that they can be used interchangeably. Our study finds that, for urine and serum concentrations of BPA and PFCs, this is not the case; the SEP measures we studied do not overlap entirely with one another, and had different estimates of effect in our regression models. We conclude that, in the context of this study, income, education, occupation, and food security do not represent the same socioeconomic constructs, but rather seem to capture different aspects of how SEP may be related to exposure to BPA and PFCs. While constraints regarding data availability and the need to maximize sample size will always be an issue, the question of which SEP measure to use is an important methodologic concern, and merits more consideration by researchers in the field.

As discussed, family income was the most important SEP predictor in our investigation. We found that adequate gradations must be used, however, to see the full extent of the effect. When modeled as a dichotomous variable with a cut point of $20,000, which is tempting to do in NHANES as there are fewer missing participants, the full effect of SEP was underestimated. There was also some indication that income measures that adjusted for household size, such as adjusted family income and PIR, were stronger predictors. Regarding education, we found that using a dichotomous variable with a cut point of high school graduation did not fully capture the SEP difference. Participants with some college or an associate's degree were more similar to high school graduates than college graduates. A disadvantage of using education as a measure of SEP is that it is not a useful measure for children, a concern that also applies to occupation. In addition, our findings related to occupation are limited due to the smaller sample size, but it may be the case that a different approach to categorizing occupation, such as one based on type of industry, would be more closely related to the outcomes of interest. Food security, though not as commonly used to assess SEP, revealed important information about a vulnerable population - children whose families have very low food security or receive emergency food aid - information that other SEP measures failed to provide.

Regarding the measurement of race and ethnicity in studies such as these, our findings show that useful information can be gleaned from considering country of origin, particularly for Mexican Americans. This follows previous examples [40,53].

Inherent in our study are a number of limitations. One concern is possible confounding by geography, which we cannot assess with publicly-available NHANES data. Regional and local populations vary in measures of SEP and race/ethnicity; if BPA and PFC exposures also differ with geography, there could be confounding. This geographic variation in chemical exposures could occur through differences in environmental contamination-localized contamination with certain PFCs has been reported in the USA (e.g., Hoffman et al. [55])- or in consumption patterns of foods and other products that lead to exposure. In particular, the striking findings for Mexican Americans must be taken with caution. Zota et al. [40] received permission to access state-level geographic information for NHANES 2003-2004 PBDE data and showed that, because Californians overall had higher serum concentrations of PBDEs and a large proportion of Mexican Americans sampled by NHANES lived in California, residence in California confounded results for Mexican Americans. Further investigation of geographical differences in body burdens would be greatly aided by the public release of NHANES data indicating region of the USA, something that would appear unlikely to threaten confidentiality.

We relied on a single biomonitoring measurement of the chemicals of interest. This is less of an issue for PFCs, which have long half-lives and we would not expect concentrations to vary significantly within an individual. For BPA, however, this is more of a concern. Mahalingaiah et al. [56] showed a single spot urine sample to be predictive of exposure over weeks to months, despite within person variability. However, they assessed a single sample's ability to classify participants into tertiles, which is not how we modeled our data. And, they concluded that a second sample offered improvements in classifying individuals.

For both compounds, there are complications involved in interpreting results from biomonitoring data. While biomonitoring measurements provide a useful estimate of internal dose, there is likely inter- and intra-individual variation in measurements as a result of various factors that influence the chemical's pharmacokinetics, i.e. its distribution among compartments of the body, metabolism, and excretion [57]. These factors include genetics, biological characteristics such as gender, body fatness, and liver function, and environmental factors such as diet, all of which may affect a chemical's pharmacokinetics. Though these concerns may be particularly relevant for BPA, which is measured as a urinary metabolite, many questions remain about the pharmacokinetic behavior of both BPA and PFCs in the body.

The measures of SEP we studied are all based on self-reported data. Getting participants to report personal income in particular is notoriously difficult [28]. However, the NHANES approach of asking people to report in income categories seemed to work reasonably well, as less than 4% of participants were missing family income data. A limitation in our assessment of SEP was the availability of only two years of data for occupation.

Our study has several strengths, including a large sample size, unrivalled in studies of this nature that involve costly biomonitoring measurements. The NHANES sampling methodology of oversampling certain racial/ethnic, income, and age groups was critical in providing an excellent distribution of participants across different categories. Thus, we had ample power to detect associations between different SEP and racial/ethnic groups, and were able to consider modification by age and gender. Another key advantage was the availability of robust data on a variety of SEP measures. This enabled us to compare different SEP-related variables.

This paper has primarily explored associations between body burdens and measures of SEP and race/ethnicity. More research is needed on the specific aspects of diet, consumer products, and other activities or circumstances that provide the links between SEP/race/ethnicity and body burdens (Figure 1). This question might be approached using dietary intake data, measures of indoor exposure, and other techniques.

## Conclusions

Characterizing social disparities in exposure to potentially harmful chemicals is an important responsibility of environmental health. The juxtaposition of BPA and PFCs together reveals a striking opposite pattern of associations with measures of SEP, particularly income. BPA levels were inversely associated and PFC levels positively associated with family income. Differences by race/ethnicity - most notably, markedly lower concentrations in Mexican Americans for both BPA and PFCs - were independent of SEP. We conclude that income, education, occupation, and food security represent distinct facets of social stratification and are not necessarily interchangeable as measures of SEP in environmental epidemiology studies. In these data, family income with adjustment for family size was the strongest predictor of BPA and PFC levels among the different measures of SEP we studied. Examining differences in body burden by a range of SEP measures can provide useful insights about the conceptual basis for the choice of SEP measure and about vulnerable populations, and can raise hypotheses about possible sources of exposure.

## Abbreviations

BPA: Bisphenol A; CDC: Centers for disease control and prevention; CI: Confidence interval; EDC: Endocrine disrupting chemical; LOD: Limit of detection; NHANES: National health and nutrition examination survey; PBDE: Polybrominated diphenyl ether; PC: Polycarbonate plastic; PFC: Polyfluoroalkyl chemical; PFHxS: Perfluorohexane sulfonic acid; PFNA: Perfluorononanoic acid; PFOA: Perfluorooctanoic acid; PFOS: Perfluorooctane sulfonic acid; SEP: Socioeconomic position; SES: Socioeconomic status; TC: Total cholesterol.

## Competing interests

The authors declare that they have no competing interests.

## Authors' contributions

JN conceived of and coordinated the study, performed the analysis, and drafted the manuscript. MKS, EH, and TW participated in study design, data analysis, and manuscript preparation. All authors read and approved the final manuscript.

## Supplementary Material

Additional file 1 **Categories of longest-held occupation**. Click here for file

Additional file 2 **Determination of final sample size for analyses**. Click here for file

Additional file 3 **Relationship of different SEP measures to adjusted family income**. Click here for file

Additional file 4 **Percent change in BPA and PFC concentrations by income and food security, stratified by age**. Click here for file
