# Supplementary figures and images for: Social disparities in exposures to bisphenol A and polyfluoroalkyl chemicals: a cross-sectional study within NHANES 2003-2006
Source: Environ Health. 2012 Mar 6;11:10. doi: 10.1186/1476-069X-11-10 (PMC3312862; doi:10.1186/1476-069X-11-10)

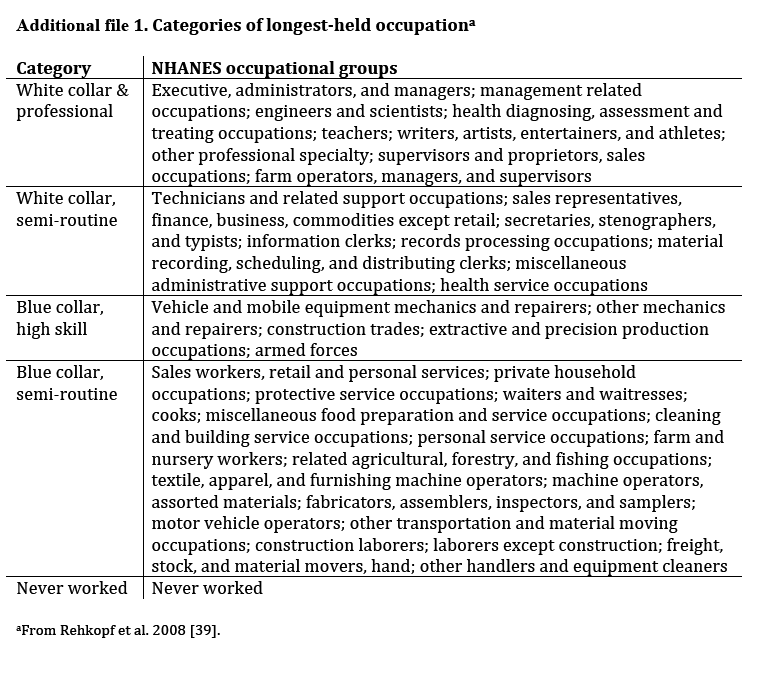

Supplement: Additional file 1 — Categories of longest-held occupation. [file 1476-069X-11-10-S1.BMP]

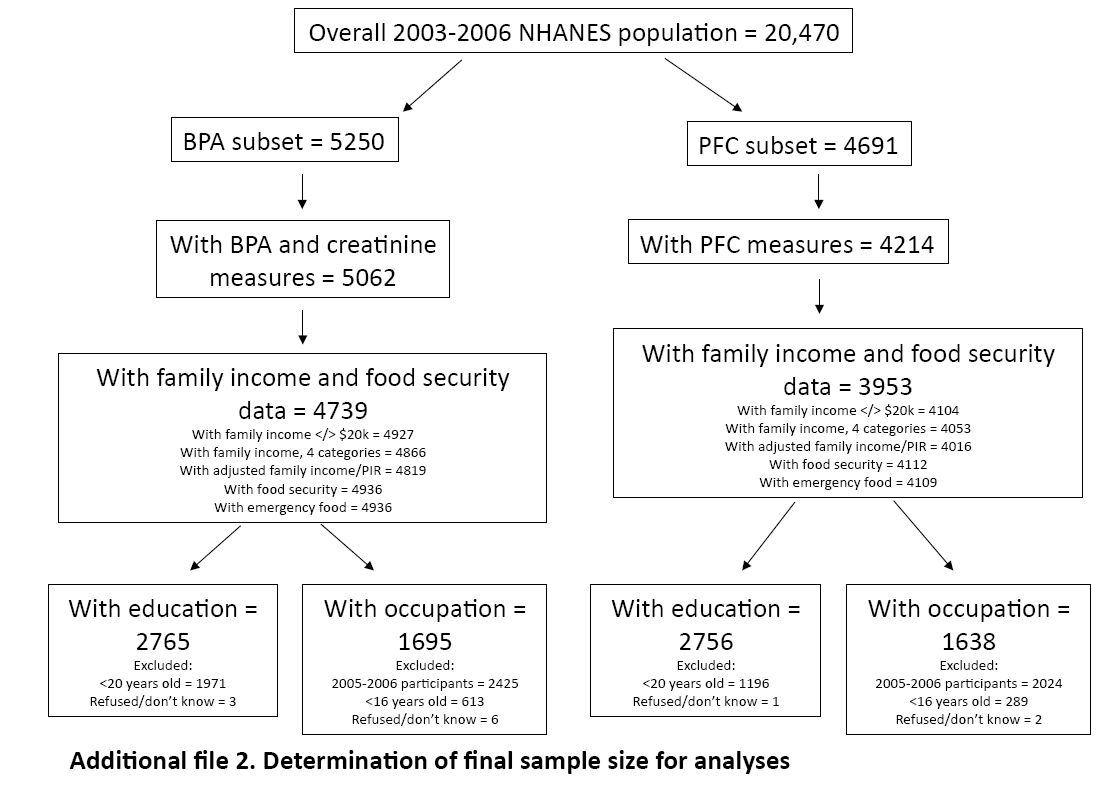

Supplement: Additional file 2 — Determination of final sample size for analyses. [file 1476-069X-11-10-S2.BMP]

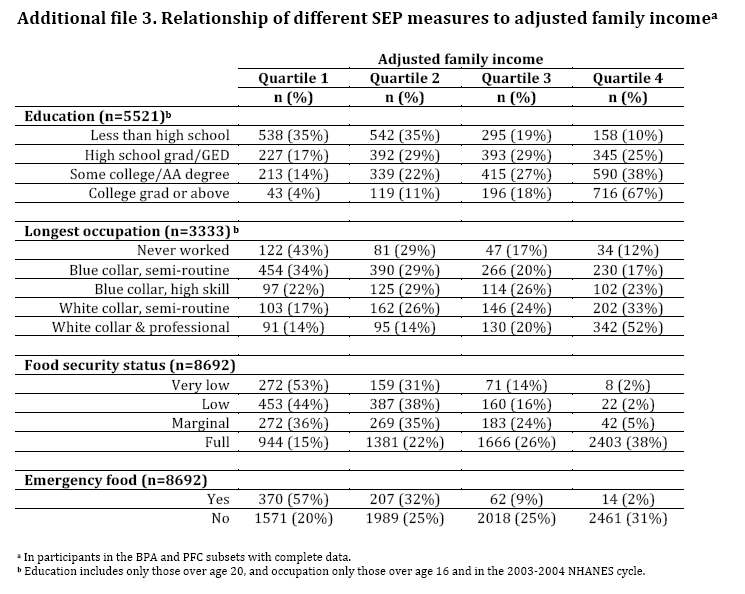

Supplement: Additional file 3 — Relationship of different SEP measures to adjusted family income. [file 1476-069X-11-10-S3.BMP]

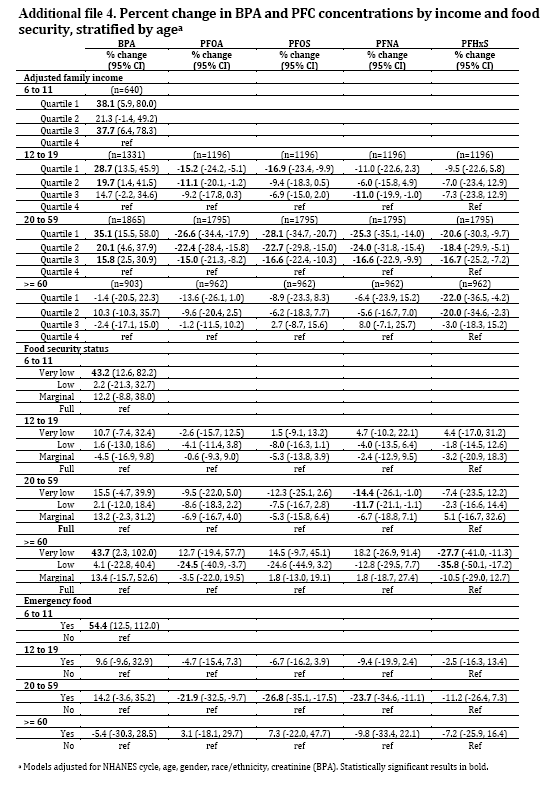

Supplement: Additional file 4 — Percent change in BPA and PFC concentrations by income and food security, stratified by age. [file 1476-069X-11-10-S4.BMP]
